# Data of vertical and horizontal handover on video transmission in Proxy Mobile IPv6

**DOI:** 10.1016/j.dib.2019.104736

**Published:** 2019-11-04

**Authors:** Md Mahedi Hassan, Ian K.T. Tan, Timothy Tzen Vun Yap

**Affiliations:** a Multimedia University, 63100, Cyberjaya, Selangor, Malaysia; b Monash University Malaysia, Bandar Sunway, 47500, Subang Jaya, Selangor, Malaysia

**Keywords:** Horizontal handover, Vertical handover, Video transmission, Wireless mobility, Seamless, Performance metrics, Data

## Abstract

The Internet Engineering Task Force provides a network-based mobility management solution to execute handover in heterogeneous networks on network-side called Proxy Mobile IPv6 (PMIPv6). In this data article, data are presented during the horizontal and vertical handover on video communication in PMIPv6 mobility protocols. The handover data are gathered using several measurement factors, which are latency, jitter, cumulative measured, and peak signal noise ratio under network simulation software, for both horizontal and vertical handovers [8].

**Table:** Specifications Table

Subject	Wireless communication
Specific subject area	The data are of the handover performance metrics of video transmissions over Proxy Mobile IPv6.
Type of data	Table Graph Figure Text Files Videos
How data were acquired	Data are obtained from video transmission during horizontal and vertical handover under simulation scenarios. Using real video data as input for the simulation, the performance metrics were extracted from the simulation tools.
Data format	Analyzed Raw
Parameters for data collection	Network Parameters: i) Transmission Rate: 384Kbps (UMTS) and 11Mbps (Wi-Fi) ii) Link Delay: 15 ms (UMTS) and 15 ms (Wi-Fi) iii) Distance of coverage: 50 m (Wi-Fi) iv) Uplink bandwidth: 384Kbps (UMTS) v) Uplink transmission time interval: 20 ms (UMTS) vi) Video traffic type: MyUDP (Both) Video Parameters: i) Video Packet Size: 1024 bytes ii) Maximum Video Fragment Size: 1000 bytes iii) Frame rate: 30 fps iv) Video Resolutions: 352 × 288, 512 × 288, 640 × 360
Description of data collection	i) Convert the actual video clips into YUV and encode into m4v format ii) Produce a file of MP4 extension that comprises the samples (frames) of video and a hint track that defines how to packetize the frames for the flow of packet iii) Create and upload the trace file into the simulation scenarios and execute the simulation scripts that produce the simulated sending and receiving time of each packet iv) Generate the performance metrics data and a video file with degraded frames over the wireless network, including all degraded frames that were lost, corrupted, and deleted from the original video track
Data source location	Multimedia University, Cyberjaya, Malaysia
Data accessibility	Repository name: Data-in-Brief (Videos and Handover Data) Direct URL to data: https://data.mendeley.com/datasets/24636wx22f/2 Hassan, Md. Mahedi; Tan, Ian K T; Yap, Timothy Tzen Vun (2019), “Handover Simulation Data on Video Transmission in Proxy Mobile IPv6”, Mendeley Data, V2, https://doi.org/10.17632/24636wx22f.2
Related research article	Md Mahedi Hassan, Ian KT Tan, Bhawani Selvaretnam, Kuan Hoong Poo SINR-based conversion and prediction approach for handover performance evaluation of video communication in Proxy Mobile IPv6 Computers & Electrical Engineering https://doi.org/10.1016/j.compeleceng.2019.01.008

**Table:** 

**Value of the Data** • The average of horizontal and vertical handover data provided in this article will facilitate empirical research in wireless mobility on video transmission in network-based mobility management protocols. • These handover data are useful for the formulation, simulation, and evaluation of mobility management protocols. Each of the performance metrics data is essential for improving the process of handover and quality of service. • Telecommunication research in wireless mobility relies on these types of data, which include handover latency, cumulative jitter for handover initiation, handover decision and handover execution. For the quality of service on video transmission, cumulative measured and peak signal noise ratio data are useful to check the performance of video quality. • The average of the total value for the performance metrics during the handover are presented in tables, making data interpretation much easier for technical conclusions. • Data shared in this data article that will open doors for potential future research endeavors and collaborations.

## 1 Data

Wireless networks and multimedia technologies have experienced significant growth in the last two decades. The use of handheld devices and obtaining services offered by the Internet has now become essential in our daily lives. Therefore, the availability of wireless networks and network quality of service (QoS) offered have become vital for mobile users. When a mobile host (MH) changes its point of attachment (access point, base station) to the same network or a new network, the availability of the wireless network becomes an essential consideration. The changing point of attachments will involve two types of shifting process; these are the horizontal handover and vertical handover [1]. When a MH shift from one access point (AP) to another, such as Wi-Fi→Wi-Fi or UMTS→UMTS, the shifting process will perform a horizontal handover [[1], [2], [3]]. Vertical handover is performed when a MH moves from one base station (BS) to an AP or another BS technology such as UMTS→Wi-Fi, LTE→WiFi or UMTS→LTE→Wi-Fi [[1], [2], [3]]. During the process of handover, the wireless connection will be lost if a MH takes a longer time to attach the new attachment point. As a result, the performance of multimedia streaming such as video transmission, voice over IP, or file downloads will degrade [[2], [3], [4],^6^].

This data article presents the video transmission data on horizontal and vertical handover in Proxy Mobile IPv6 (PMIPv6) [[2], [3], [4], [5], [6]]. The data are measured using average performance metrics, which are the packet latency, frame latency, cumulative jitter, cumulative measured, and peak signal noise ratio (PSNR) [7]. The data are provided with two types of handover scenarios, one involving just one MH and the other involving three MHs.

### 1.1 Horizontal handover data

**Fig. 1:**
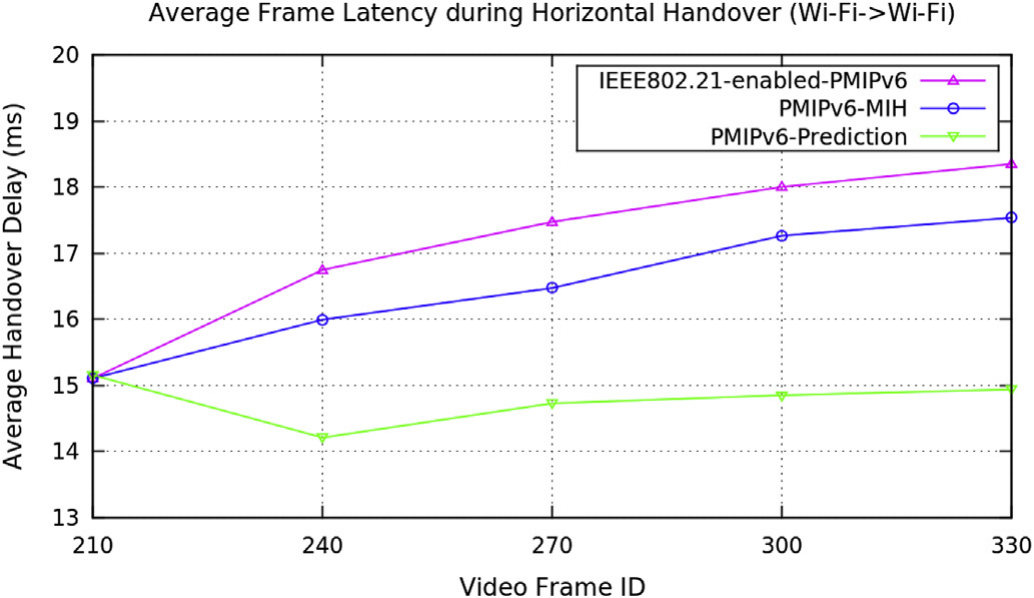
Average frame delay during horizontal handover (Avengers-2 video clip).

**Fig. 2:**
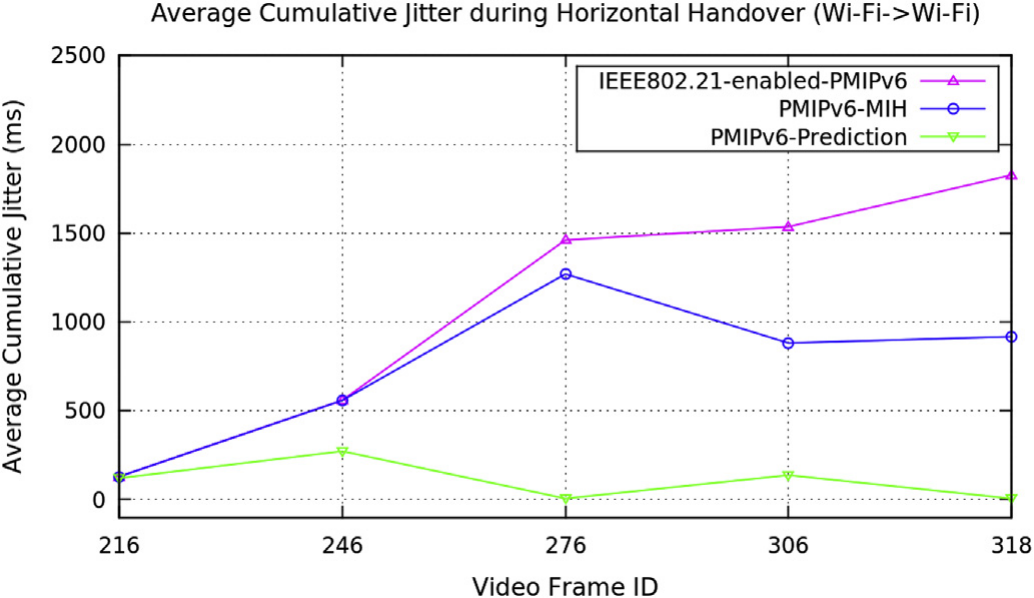
Average frame cumulative jitter during horizontal handover (Avengers-2 video clip).

**Fig. 3:**
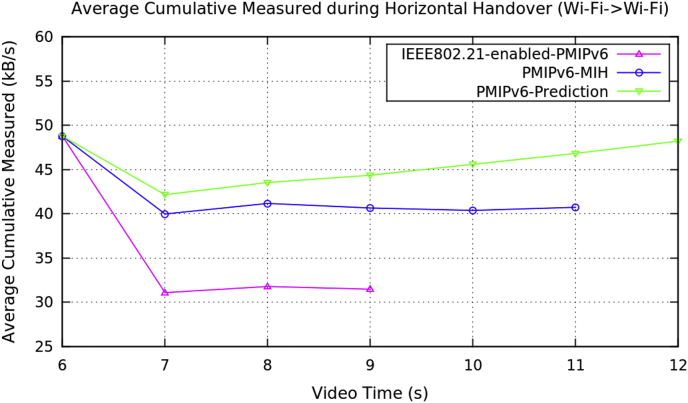
Average frame cumulative measured during horizontal handover (Avengers-2 video clip).

**Fig. 4:**
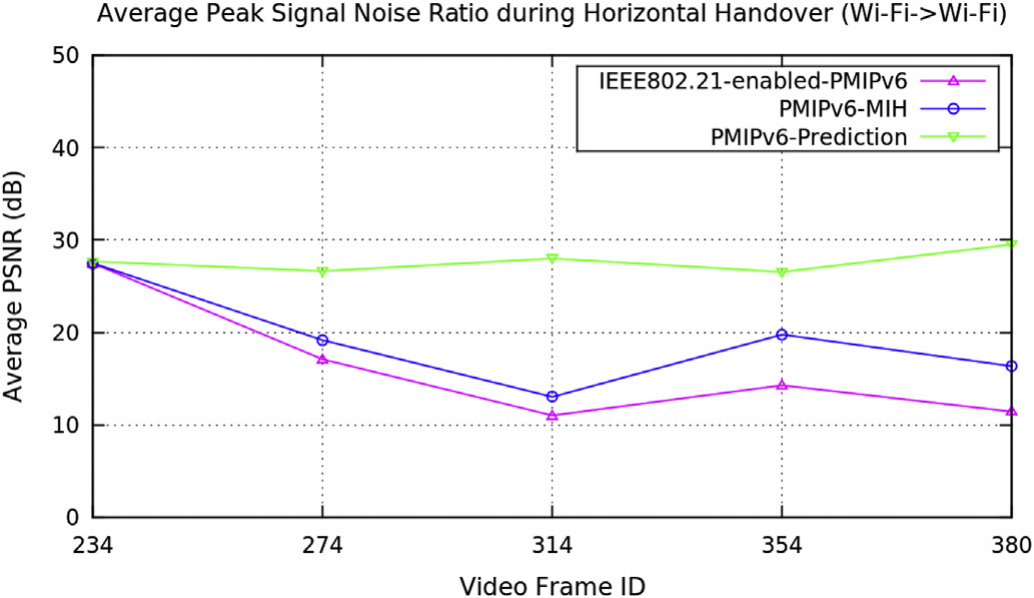
Average peak signal noise ratio during horizontal handover (Avengers-2 video clip).

Average performance analysis of the horizontal handover data on video transmission along the three mobility protocols of the PMIPv6 is presented in Table 1. The three mobility protocols of the PMIPv6 are PMIPv6-Prediction [8], PMIPv6-MIH [9] and IEEE802.21-enabled-PMIPv6 [10]. Fig. 1 depicts the performance metrics of average handover latency, Fig. 2 depicts the cumulative jitter, Fig. 3 depicts the cumulative measured, and Fig. 4 depicts the PSNR of a video frame during the horizontal handover of video transmissions for the three mobility protocols of the PMIPv6.

**Table 1:** Average data of performance metrics during horizontal handover in PMIPv6.

Average Performance Metrics During Horizontal Handover	Mobility Protocols of Proxy Mobile IPv6		
	PMIPv6-Prediction	PMIPv6-MIH	IEEE802.21-enabled-PMIPv6
Frame Handover Latency (ms)	15.153	15.107	15.107
	14.207	15.994	16.748
	14.727	16.474	17.475
	14.85	17.266	18.004
	14.936	17.537	18.351
Frame Cumulative Jitter (ms)	120.983	129.576	129.856
	272.338	558.64	558.92
	6.439	1270.08	1461.14
	137.642	881.215	1536.06
	5.90693	917.268	1826.89
Cumulative Measured (kB/s)	48.7769	48.767	48.7689
	42.1612		
	43.5323	39.9754	
	44.3586	41.1685	
	45.595	40.6579	31.0779
	46.8263	40.3813	31.7759
	48.2176	40.7358	31.4691
Peak Signal Noise Ratio (dB)	27.7	27.49	27.47
	26.6446	19.1522	17.0861
	28.0076	13.0146	11.0073
	26.5541	19.7674	14.2669
	29.5299	16.3445	11.4209

Average handover delay of the video frame determines the period after an MH sends packets from its present position to a new position. These can be from AP to AP, or from BS to AP, or from one network to another network, as long as the updated frame allows access to the respective networks.

### 1.2 Vertical handover data

**Fig. 5:**
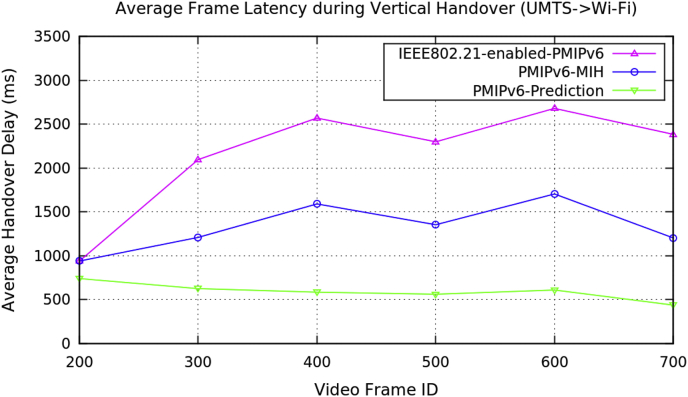
Average frame handover delay during vertical handover (Avengers-2 video clip).

**Fig. 6:**
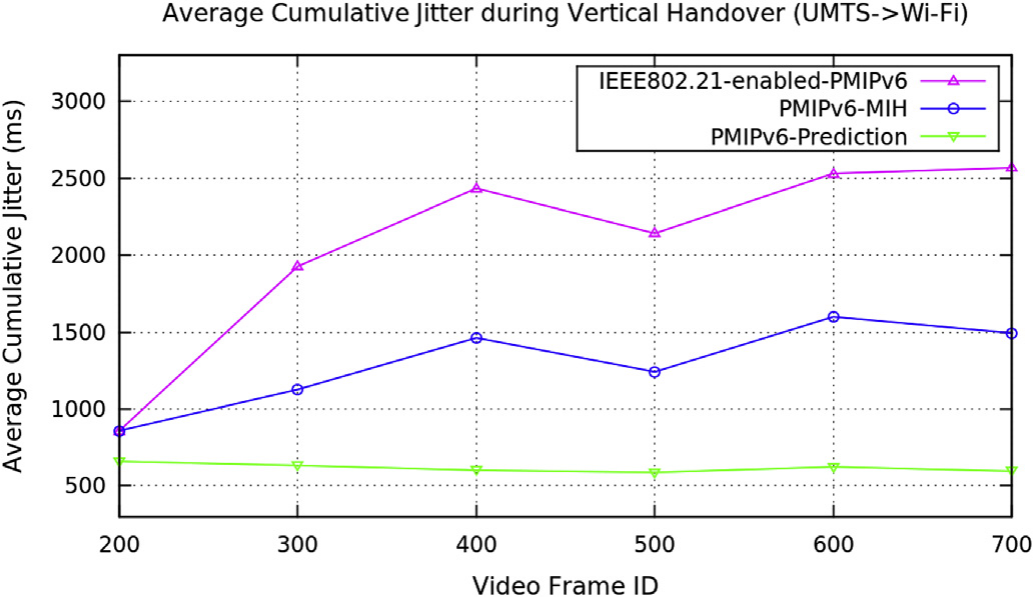
Average frame cumulative jitter during vertical handover (Avengers-2 video clip).

**Fig. 7:**
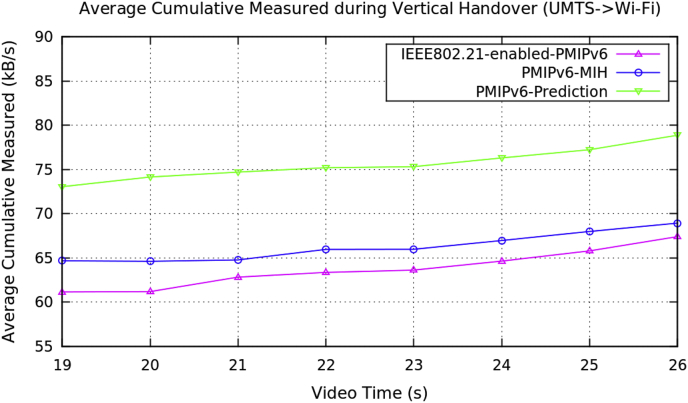
Average frame cumulative measured during vertical handover (Avengers-2 video clip).

**Fig. 8:**
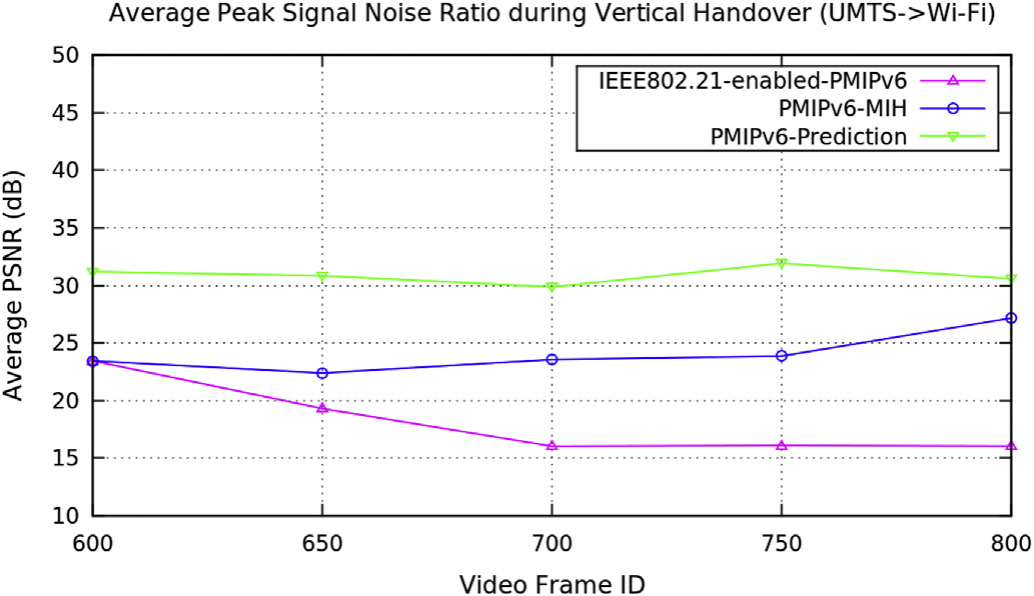
Average peak signal noise ratio during vertical handover (Avengers-2 video clip).

Table 2 represents the average performance data for the vertical handover of video transmission with the three PMIPv6 mobility protocols. Fig. 5, Fig. 6, Fig. 7, and Fig. 8 show the performance metrics of average handover latency, cumulative jitter, cumulative measured and PSNR of video frame during vertical handover on video transmission in PMIPv6 mobility protocols. During the vertical handover on video transmission, the mobility protocols of PMIPv6-MIH and IEEE802.21-enabled-PMIPv6 have increased latency and jitter, therefore causing a degradation of video transmission performance. This is because these protocols are not designed to decide on the necessary handover conversion as they lack the essential information in the protocols.

**Table 2:** Average data of performance metrics during vertical handover in PMIPv6.

Average Performance Metrics During Horizontal Handover	Mobility Protocols of Proxy Mobile IPv6		
	PMIPv6-Prediction	PMIPv6-MIH	IEEE802.21-enabled-PMIPv6
Frame Handover Latency (ms)	739.099	939.099	939.099
	625.187	1209.19	2096.19
	584.857	1590.8	2570.01
	561.238	1353.24	2299.24
	609.425	1704.47	2680.59
	436.759	1202.57	2384.62
Frame Cumulative Jitter (ms)	659.034	859.034	859.034
	633.122	1126.12	1928.12
	601.821	1462.22	2434.49
	587.673	1240.67	2142.17
	624.018	1599.14	2531.48
	595.743	1493.35	2567.84
Cumulative Measured (kB/s)	73.0548	64.677	61.1518
	74.1486	64.6144	61.1892
	74.7004	64.7721	62.8229
	75.204	65.9635	63.3583
	75.3153	65.9734	63.6186
	76.3107	66.9669	64.6403
	77.2351	67.9886	65.7921
	78.8777	68.9279	67.4041
Peak Signal Noise Ratio (dB)	31.2	23.44	23.44
	30.8384	22.3826	19.2946
	29.8653	23.5559	16.0241
	31.92	23.8603	16.0891
	30.5796	27.1602	16.0346

### 1.3 Vertical handover data with 3 concurrent videos

**Fig. 9:**
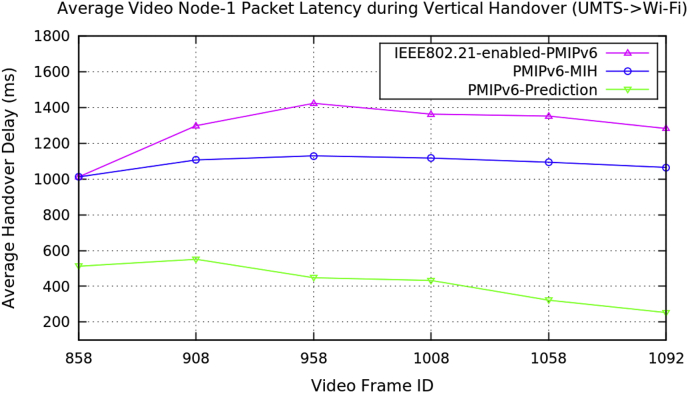
Average video Node-1 packet latency during vertical handover (baby boss video clip).

**Fig. 10:**
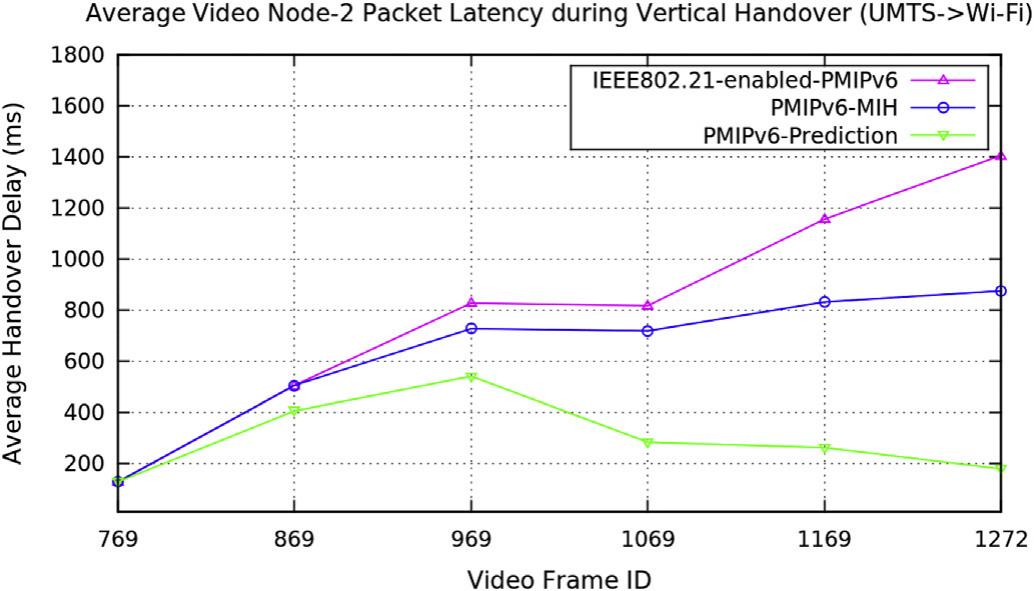
Average video Node-2 packet latency during vertical handover (Transformers-4 video clip).

**Fig. 11:**
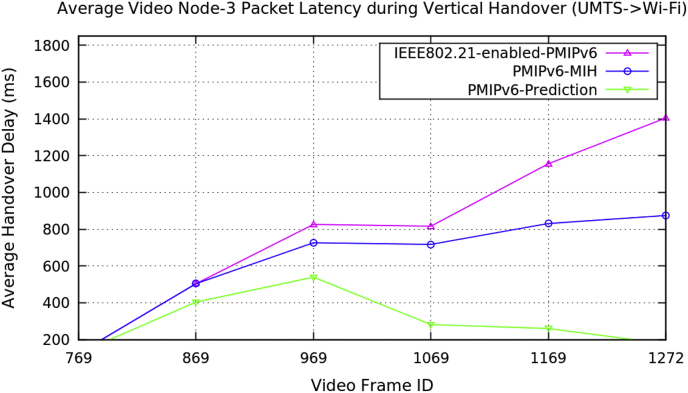
Average video Node-3 packet latency during vertical handover (minions video clip).

Fig. 9, Fig. 10, and Fig. 11 illustrate the performance metric of average packet latency of each packet (Packet ID) during the vertical handover on video transmission in PMIPv6 mobility protocols. Table 3 represents the data for the average packet latency during the vertical handover of three video nodes in PMIPv6 mobility protocols.

**Table 3:** Average data of three video nodes performance metrics during vertical handover in PMIPv6.

Average Performance Metrics During Vertical Handover	Mobility Protocols of Proxy Mobile IPv6		
	PMIPv6-Prediction	PMIPv6-MIH	IEEE802.21-enabled-PMIPv6
Packet Handover Latency (ms) Video Node-1 Baby Boss Video Clip Video Size: 640 × 360	512.101	1012.1	1012.1
	551.112	1107.11	1299.11
	447.718	1130.07	1424.19
	432.528	1117.53	1363.53
	322.31	1094.59	1353
	252.749	1065.34	1283.12
Packet Handover Latency (ms) Video Node-2 Transformers-4 Video Clip Video Size: 512 × 288	126.123	126.123	126.123
	404.414	504.414	504.414
	539.984	726.949	826.949
	281.932	717.923	816.923
	260.544	831.578	1156.38
	177.101	874.879	1405.51
Packet Handover Latency (ms) Video Node-3 Minions Video Clip Video Size: 510 × 288	413.108	413.108	413.108
	796.869	796.869	796.869
	957.29	1057.29	1257.29
	982.36	1060.36	1260.36
	1170.03	1285.71	1416.34
	476.078	1070.69	1448.11

## 2 Experimental design, materials, and methods

Experiments are conducted on video transmissions during the handover in PMIPv6 mobility protocols using network simulation software [11]. The data provided here are from two types of mobility simulation scenarios, which are horizontal and vertical handover. The simulation scenarios that resulted in the data are presented in Table 1, Table 2, Table 3 and are illustrated in figures published by Hassan et al. [8].

The EvalVid video simulation package is utilized for the video transmission simulation, where the MPEG-coded video stream is defined as a source model for MPEG4 traffic [12,13]. The video size used is Common Intermediate Format (CIF) or H.261 which has a resolution of 352 × 288 [14]. In this simulation, a video clip is converted to the CIF format from the movie “Avengers: Age of Ultron” [8].

Three video nodes are set up with two different videos with two frame sizes, which are 640 × 360 and 512 × 288 [8]. In this simulation, three different video clips are converted to the MPEG4 format which are video node-1, video node-2 and video node-3. The videos are from “The Baby Boss”, “Transformers: Age of Extinction” and “Minions” respectively [8]. Video node-1 (MH1) is set up with 640 × 360 frame size and video node-2 and video node-3 are set up with 512 × 288 frame size. The video packet size is set up for 1024 bytes whereas the distance between consecutive packets is set at 0.001 seconds.

**Fig. 12:**
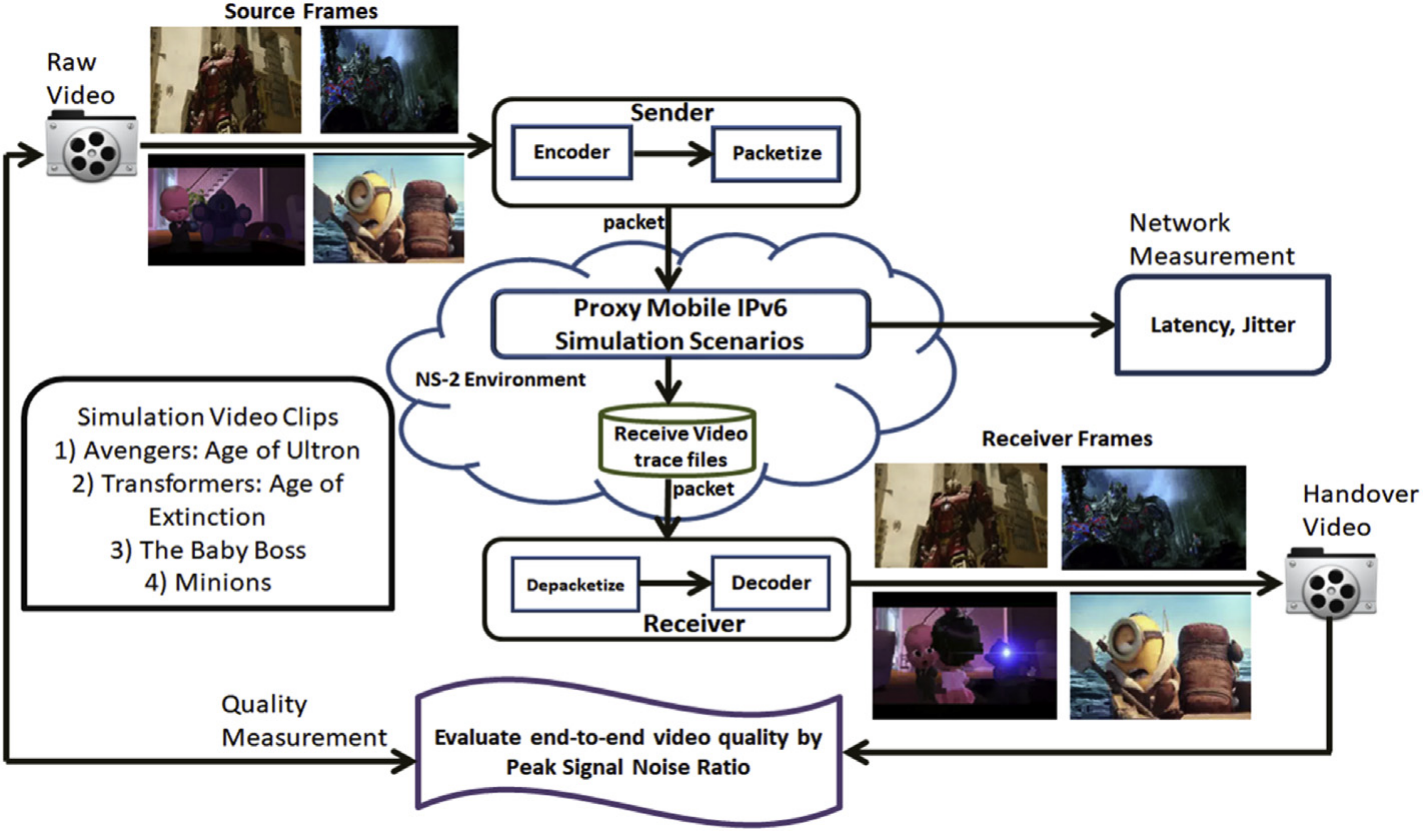
Handover data collection of simulation scenario of PMIPv6-Prediction, PMIPv6-MIH, IEEE802.21-enabled-PMIPv6.

The process of data collection is shown in Fig. 12. The videos data are converted into YUV format to produce the packetized data for the sender. These packetized data are installed in the PMIPv6 simulation scenarios to collect packetized data at the receiver side. Upon receiving the packetized data, handover data are collected and converted into YUV format for receiver video output.

Table 4, Table 5, Table 6 represent the average total value of performance metrics during horizontal and vertical handovers in PMIPv6 mobility protocols. The handover performances are presented in the total value of the average frame and packet metrics (in millisecond). The metrics are handover latency and cumulative jitter. The quality of performances is presented in the total value of the average frame in kilobytes per second and decibel, for the cumulatively measures and PSNR respectively.

**Table 4:** Average of total performance metrics during horizontal handover in PMIPv6.

Average of Total Value of Performance Metrics During Horizontal Handover	Mobility Protocols of Proxy Mobile IPv6		
	PMIPv6-Prediction	PMIPv6-MIH	IEEE802.21-enabled-PMIPv6
Frame Handover Latency (ms)	14.77	16.47	17.13
Frame Cumulative Jitter (ms)	108.66	751.35	1102.57
Cumulative Measured (kB/s)	45.21	41.95	35.77
Peak Signal Noise Ratio (dB)	27.68	19.15	16.25

**Table 5:** Average of total performance metrics during vertical handover in PMIPv6.

Average of Total Value of Performance Metrics During Vertical Handover	Mobility Protocols of Proxy Mobile IPv6		
	PMIPv6-Prediction	PMIPv6-MIH	IEEE802.21-enabled-PMIPv6
Frame Handover Latency (ms)	276.39	537.39	825.49
Frame Cumulative Jitter (ms)	616.90	1296.76	2077.19
Cumulative Measured (kB/s)	75.60	66.23	63.74
Peak Signal Noise Ratio (dB)	31.08	23.67	18.17

**Table 6:** Average of total packet latency during vertical handover in PMIPv6.

Average of Total Value of Packet Latency During Vertical Handover	Mobility Protocols of Proxy Mobile IPv6		
	PMIPv6-Prediction	PMIPv6-MIH	IEEE802.21-enabled-PMIPv6
Video Node-1 (Video Size: 640 × 360) (ms)	419.75	1087.79	1289.17
Video Node-2 (Video Size: 512 × 288) (ms)	322.59	630.31	806.05
Video Node-3 (Video Size: 512 × 288) (ms)	799.28	947.33	1098.68
